# Whole exome sequencing in a juvenile idiopathic arthritis large family with SERPINA1 gene mutations

**DOI:** 10.1186/s41927-022-00269-9

**Published:** 2022-07-04

**Authors:** Cyprian Popescu

**Affiliations:** Clinic Victor Pauchet, Amiens, France

**Keywords:** Juvenile idiopathic arthritis, Psoriasis, Alpha-1-antitrypsin deficiency (AATD), SERPINA1

## Abstract

**Objectives:**

Although the underlying mechanisms and mediators of arthritis in juvenile idiopathic arthritis are not well understood, accumulated evidence supports the mixt role of genetic and environmental factors. Few reports of multiplex families with JIA were published until now. The aim of this study was to describe the subjects affected by juvenile idiopathic arthritis and psoriatic features (JIAPs) in a large family.

**Methods:**

Here, we characterized an extended multiplex family of 5 patients with juvenile idiopathic arthritis and psoriatic features (PsA) at the clinical and genetic level, using whole exome sequencing.

**Results:**

We did not confirm in our family the linkage with the genetic factors already described that might be associated with increase susceptibility to JIA. We found a carrier status of siblings who inherited a pathogenic allele of the SERPINA1 gene from their mother who herself has two heterozygous pathogenic variants in the SERPINA1 gene.

**Conclusions:**

This study didn’t identify genetic contributive factors but highlights potentially environmental associations concerning the siblings of a family with juvenile idiopathic arthritis and psoriatic features (JIAPs). It is difficult to establish that SERPINA1 gene mutation has an etiological role as the levels of AAT are only slightly decreased and all the children harbor heterozygous variants.

## Introduction

Juvenile idiopathic arthritis (JIA) is a juvenile-onset childhood rheumatic disease with heterogenic phenotypes. JIA may result from an interplay of genetic, epigenetic, and environmental risk factors. Psoriatic juvenile idiopathic arthritis (JIAPs) is usually seronegative for rheumatoid factor and is one of the seven subtypes according to the Pediatric Task Force of the International League of Associations for Rheumatology (ILAR) [1]. Phenotypic overlap exists between these subtypes of JIA which could be the result of shared genetic/epigenetic factors [2]. JIA has similarities with other autoimmune diseases demonstrated by genome-wide association studies which identified overlapping regions between JIA and other autoimmune diseases [3]. Examination of extended multiplex families in linkage studies is a strategy to identify other genetic factors that predispose to JIA. Here, we characterized a large family of 5 patients with juvenile idiopathic arthritis and psoriatic features who harbor pathogenic variants in the SERPINA1 gene.

## Methods

The present study enrolled 5 patients from the same family with JIAPs referred for genetic diagnostic by whole exome sequencing (WES) to (Rostock, Germany) Centogene AG. DNA was extracted from salivary samples and exome capture was carried out with the method described before [4]. All inheritance patterns were considered, and advanced genotype–phenotype are used to evaluate identified variants concerning their pathogenicity and causality. The generated library sequenced on an Illumina platform ensured a sequencing coverage level of at least 20× targeting for more than 98% of the human coding exome. All variants with minor allele frequency (MAF) of less than 1% in gnomAD database, and disease-causing variants reported in HGMD®, in ClinVar or in CentoMD® are considered. Allele’s frequency was based on Genome Aggregation Database (gnomAD), Exome Sequencing Project (ESP), 1000Genome project (1000G) and CentoMD®. Low-quality single nucleotide variants and all relevant deletion/insertion variants are confirmed by Sanger sequencing. The study was performed in accordance with the modified version of the Helsinki declaration. Written informed consent to participate and publish this study was obtained from the index-case and parents of the young patients.

## Results

**Fig. 1:**
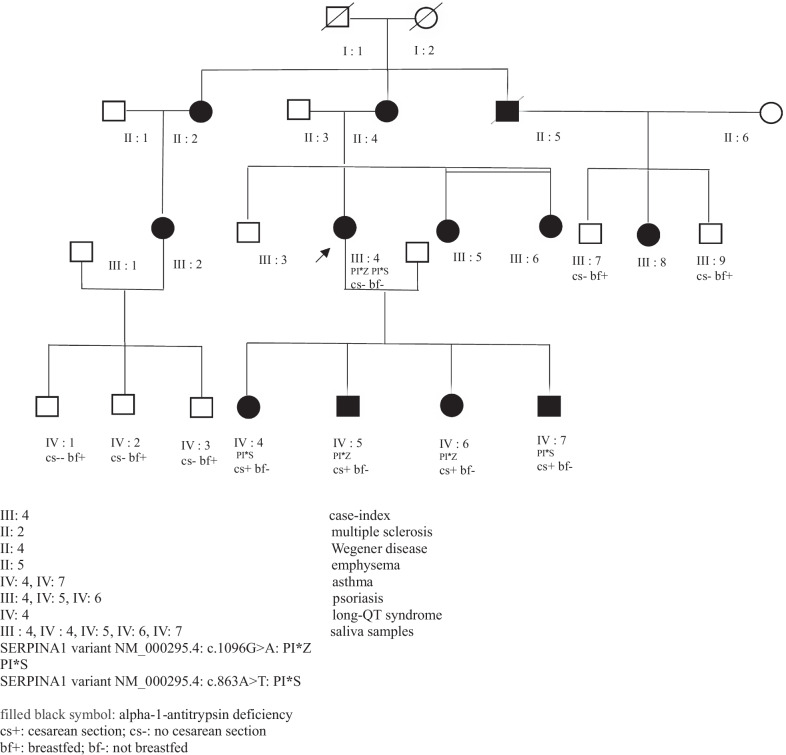
Clinical presentation of a juvenile idiopathic arthritis family with SERPINA1 gene mutations

In the present study, we investigated an association between JIAPs and SERPINA1 mutations which have been shown to be linked with autoimmune and inflammatory diseases. The patients from white French rural populations without consanguineous marriages identified with JIAPs and/or alpha-1 antitrypsin deficiency (AATD) are shown in Fig. 1. Clinical and biological features are presented in Table 1.

**Table 1:** Clinical and biological features of a juvenile idiopathic arthritis family

Patients	III: 4	IV: 4	IV: 5	IV: 6	IV: 7
Age	40	20	17	16	13
Age of onset	8	11	8	10	8
Cesarean	N	Y	Y	Y	Y
Birth weight	2800	2700	3320	3340	3370
Breastfeeding ≥ 6 months	N	N	N	N	N
Maternal smoking during pregnancy	N	N	N	N	N
Passive smoking	N	N	N	N	N
Living in a rural area	Y	Y	Y	Y	Y
Living on a farm during first year of life	N	N	N	N	N
Household pets	Y	Y	Y	Y	Y
Periodontitis/rhinitis	N	N	N	N	N
Antibiotics exposure	N	N	N	N	N
Psoriasis	Y	N	N	Y	N
Asthma	Y	Y	N	N	Y
Serum alpha-1 antitrypsin	0.59 g per liter	0.99 g per liter	0.78 g per liter	0.68 g per liter	0.97 g per liter

*Y* Yes, *N* No

III: 4, the case-index, 40 years, had the onset at 8 years, at the entry to primary school, with back pain, talalgias, interphalangeal digital arthritis and psoriasis (scaling skin, scales on the scalp and pruritus). Physician global assessment (PGA) and patient global assessment (PtGA) of disease activity show minimal disease activity. She has also asthma and alpha-1-antitrypsin deficiency (AATD) with a low AAT level at 0.59 g per liter. No biological therapies were administered.

IV: 4, a 20-year-old girl, the eldest daughter of index case, has juvenile arthritis with asymmetric oligoarthritic pattern without skin changes. At the age of 11 years, she experienced joint swelling in both knees. She suffers from asthma and has prolonged QT interval with notion of sudden death of her paternal grandfather at age 46*.* Currently, she has a treatment by adalimumab at a posology of 40 mg administered twice monthly. The serum alpha-1-antitrypsin (AAT) is at 0.99 g per liter.

IV: 5, a 17-year-old boy, the eldest son of index case, has polyarticular juvenile arthritis. The first manifestation was at 8 years old like a sacroiliac pain and joint effusion of the hips more severe in the right. He suffers from dactylitis of all toes, metatarsophalangeal joints, thumbs, and heels. On physical examination, he had bilateral synovial thickening of the proximal interphalangeal joints of the fingers even more in the metacarpo-phalanx of the right thumb, metatarsal, and metacarpal-phalangeal joints. Psoriasiform lesions appeared on elbows and scalp but there are no nail anomalies. One episode of uveitis was reported with no recurrences. He is not relieved by diclofenac and indomethacin therefore methotrexate at a posology of 10 mg per week used in combination with etanercept at the posology is 0.8 mg per kg was started at the age of 13. A few months later, a severe arthritis flare occurred, with an aggressive polyarticular course. He thus needed a wheelchair and an increased dose of etanercept at 35 mg per week. Dactylitis of all toes was noted on physical examination with a very painful limitation of the right hip and the impossibility to stand up. JADAS (juvenile arthritis disease activity score) is 12. The AAT level is at 0.78 g/L.

IV: 6, a 16-year-old girl was born by cesarean section like her brothers. She is followed for psoriasis lesions on elbows and scalp and psoriatic oligoarticular arthritis with the age of onset at 10 years. She has also AATD with a low AAT level at 0.68 g/L. Currently, she is completely asymptomatic and has no biological-based therapies. She was experiencing losses of consciousness and is followed by cardiologists because of a familial record of sudden death.

IV: 7, a 13-year-old boy who had his first rheumatic manifestation at 8 years. The joints were involved in an asymmetric pattern with recurrent hip arthritis, back pain, right wrist, shoulders, and right heel pain. He has high disease activity with painful swelling of the left knee, along with morning stiffness at about 30 min and a limited range of motion. Treatments by indomethacin then naproxen were not effective. Methotrexate was started at the age of 12. The evolution is unfavorable for 2 years with hips, right heel, and right wrist aches and back pain. On physical examination, there were noted arthritis of the wrists, knees, ankles, distal and proximal interphalangeal joints with typical “sausage digit”, and limited range of motion of hips at about 10° of abduction. JADAS score is 14. Considering the poor clinical control achieved with the current medications, anti-TNF alpha therapy (adalimumab), was administered subcutaneously at the dose of 40 mg twice monthly in association with the non-steroidal anti-inflammatory drugs therapy. Clinical remission was noted with a partial resolution of articular manifestations involving essentially the right hip and back. He has also asthma related to AATD and low serum concentration at 0.78 g/L.

The mother of the index-case (II: 4) has granulomatosis with polyangiitis (Wegener disease) involving the respiratory tract related to AATD. Her sister (III: 5) has an *inflammation* of the knees without skin changes. Her aunt (II: 2) has multiple sclerosis and her great uncle (II: 5) has symptomatic AATD (emphysema). In our patients’ rheumatoid factor (RF), HLA-B27, and anti-nuclear antibodies (ANAs) are negative.

In the index case (III: 4), two heterozygous pathogenic variants were identified in the SERPINA1 gene: SERPINA1, c.1096G > A p. (Glu366Lys) and SERPINA1, c.863A > T p. (Glu288Val) therefore the genetic diagnosis of autosomal recessive alpha-1 antitrypsin deficiency is likely confirmed. A heterozygous pathogenic variant (c.863A > T p. (Glu288Val) was identified in the SERPINA1 gene in patients IV: 4 and IV: 7. A heterozygous pathogenic variant was identified in the SERPINA1 gene (c.1096G > A p. (Glu366Lys) in the siblings IV: 5 and IV: 6.

## Discussion

Juvenile idiopathic arthritis (JIA) is a multifactorial disease because of a complex interplay of genetic and environmental factors [5] and constitutes a clinically heterogeneous group [6]. Psoriatic arthritis is one of the seven subcategories according to the Pediatric Task Force of the International League of Associations for Rheumatology (ILAR) [1]. Psoriatic arthritis represents only 2–15% of all JIA cases and is characterized by psoriatic lesions and/or family history in first-degree relatives [7]. The magnitude of the genetic contribution to JIA susceptibility is demonstrated by significantly higher disease prevalence in siblings with JIA than in adult rheumatoid disease [8], as high as 11.6 [9]. The population attributable risk percentage of JIA due to familial factors is rather small [10] estimated at 13% [9]. Meanwhile, the magnitude of the genetic component in monozygotic twins concordant for JIA is estimated at only 25–40% [8]. The monogenic form of JIA was noted in two monozygotic twin girls by detecting NFIL3 mutations involved in the immune system perturbations [11]. In two siblings with systemic JIA was found a homozygous mutation in the FAMIN gene also involved in the immune system regulation and promotion of oxidative stress [12] and a functional intronic variant in UNC13D disrupting an NF-κB enhancer was reported in a patient with systemic JIA and recurrent macrophage activation syndrome (MAS) [13]. Given the clinical and genetic heterogeneity, it would be necessary to examine separately the JIA categories. It will become essential as rare, highly penetrant mutations in JIA families are not usual*. *It is worth mentioning that the same genes that harbor rare, causal mutations in multiplexes families also harbor other susceptibility factors likely to account for developing the disease in outbred populations. Exome sequencing represents a valuable tool to identify rare highly penetrant mutations that segregate with the disease [14]. However, in our family, there is no evidence for the association of several genetic factors already identified [15, 16]. The variants described until now represent only a small percentage of genetic susceptibility and therefore constitute an important contingent of missing heritability. Because a lot of causal variants are yet to be discovered their effect sizes may be currently underestimated. Moreover, have not yet been identified all the risk variants which are topographically and functionally interrelated [17]. Therefore, the interest in the identification of extended multiplex families to bring to light new genetic or environmental factors with a role in the etiology of JIAPs. In this study, we characterized, a family with SERPINA 1 mutations with a variable deficiency in alpha-1 antitrypsin (AAT) and we questioned about a role in sensitizing to arthritis development in these patients. All siblings of our family were found to be heterozygous for pathogenic SERPINA 1, while their mother is compound heterozygous. It has long been purported that the effect of AAT is pleiotropic with strong anti-inflammatory properties, and we can speculate that a genetic predisposition resulting in enhancing the enzymatic degradation of normal host tissue could contribute, in a special environment, to the arthritic phenotype. While most patients with AAT deficiency (AATD) have presented with emphysema or liver disease, it is recognized that the clinical spectrum of AAT diseases is much larger ranging from asymptomatic to severe systemic disease. Three of our patients have asthma and an uncle was diagnosed with emphysema. In turn, the relevance of AATD at the etiology of JIA in the heterozygous state of children and compound heterozygous of their mother is not clear. In our family, common deficiency alleles are PI Z and PI S whereas severe AATD is PI type ZZ. Rarely was described a serious condition named necrotizing panniculitis [17] but there were no case reports concerning the rheumatological involvement of AATD. The siblings of our family even those with more severe phenotype have a serum AAT level around 80 mg/dL which is considered a threshold value of the risk of developing a severe disease [18]. However, some autoimmune diseases including rheumatoid arthritis and psoriasis were associated with exacerbated proteolysis by neutrophil elastase with a role in phagocytose of immune complexes [19]. Moreover, patients with rheumatoid arthritis exhibit a higher frequency of Pi types MZ and SZ genotypes than in the control adults but not validated in patients with JIA [20]. However, there is no functional assay proving the inflammatory consequences of all mutations described. Because only a small population-attributable risk of JIA is related to genetic factors, informative data support the environmental contribution to the disease. However, research to identify environmental risk factors has been relatively limited even if their role in susceptibility to JIA seems to be more important than genetic ones [10]. We noted in our family two important environmental risk factors: cesarean section and absence of breastfeeding, which are likely to influence the severity of the phenotype. There is now a large body of evidence suggesting that cesarean delivery may constitute a susceptibility factor of JIA. In our family, all children were born by cesarean section knowing that they have an immunological different response with reduced levels of humoral and cellular factors compared to vaginally delivered newborns [21]. The mode of delivery modulates the immune status as the cesarean section was associated with diminished microbial diversity [22]. Similar evidence has been brought regarding the mode of delivery on the composition of neonatal gut microbiota, and the maturation of the intestinal epithelium capable to control the immune system during early life [23]. Newborns delivered by cesarean may have an early microbiota alteration and consequently different patterns of the immune response [24]. Our study provides evidence that birth weight may have a limited role in the risk to JIAPs knowing that fetal growth and timing of birth may be factors of the susceptibility to JIA [25]. It has long been recognized that breastfeeding plays a role in the pathogenesis of JIA, including prevention of the development of JIA [26]. Moreover, a case–control study in a prospective birth cohort shows evidence of delaying or even prevention of JIA as all children were breast fed beyond 4 months of age as it was the case for our family [27]. A Longitudinal Observational Cohort Study of JIA patients has found an alteration of the composition of the microbiota of treatment-naive JIA patients characterized by a lower diversity and richness compared to healthy controls [28]. Short Chain Fatty Acids (SCFAs) are considered the main metabolic products of intestinal bacterial fermentation with a role in the regulation of the immune system. SCFAs have an anti-inflammatory role by reducing the pro-inflammatory factors, including TNF-α and IL-1β, and regulating several leukocyte functions [29]. There is evidence of the cooperation between linoleic acid (LA), another polyunsaturated fatty acid, and α-1 antitrypsin (AAT) against the inflammatory factors as LA stimulates AAT to inhibit lipopolysaccharide-induced IL-1β [30]. Another study suggests the possibility that breastfeeding may protect from the development of psoriatic arthritis subtype of JIA [31]. In our case, the hygiene hypothesis concerning the early exposures to infections and subsequently to protective non-pathogenic microbiota [32] was not verified as the youngest brother has the most aggressive phenotype. We did not find other environmental factors in the development of JIAPs as smoke exposure [33], allergic rhinitis, infections [34] or antibiotics exposure earlier in life [35]. In our family, living in a rural area, was a permanent cat contact since childhood. Furred pets may alter the microbial environment and therefore could sensitize to childhood allergies but along with other infectious risks early in life could be confounding factors. Moreover, contact with the farm environment in infancy might not be associated with JIA [36]. There are literature reports on earlier onset of the disease in patients from multiplex families of JIA than in sporadic cases [37] not proven in our family whose mean age of onset is close to that obtained in a cohort of sixty JIA children [38].

The main limitation of our study was that it only assessed patients from a single center. There are also technical limitations given that complex genetic events such as inversions, translocations and repeat expansions, may not be reliably detected and certain regions may be poorly covered.

## Conclusion

This study didn’t identify genetic contributive factors but highlights potentially environmental associations concerning the siblings of a kindred with juvenile idiopathic arthritis, and psoriatic features (JIAPs). It is difficult to establish that SERPINA1 gene mutation has an etiological role as the levels of AAT are only slightly decreased and all the children are heterozygous. Further investigation must be done to prove whether SERPINA1 mutations may have a potential JIA causality.

## Data Availability

The datasets used and/or analyzed during the current study are available from the corresponding author, upon reasonable request. The data are not generally accessible because their containing information could compromise the privacy of research participants.

## Abbreviations

JIA: Juvenile idiopathic arthritis
JIAPs: Juvenile idiopathic arthritis and psoriatic features
PsA: Psoriatic features
AAT: Alpha-1-antitrypsin
AATD: Alpha-1-antitrypsin deficiency
JADAS: Juvenile arthritis disease activity score
SCFAs: Short chain fatty acids
